# Assessment of Range of Movement, Pain and Disability Following a Whiplash Injury

**DOI:** 10.2174/1874325001711010541

**Published:** 2017-07-19

**Authors:** Atif A. Malik, Simon Robinson, Wasim S. Khan, Bernice Dillon, Martyn E. Lovell

**Affiliations:** University College London Institute of Orthopaedics and Musculoskeletal Science, Royal National Orthopaedic Hospital, Stanmore, Middlesex, London; HA7 4LP, UK

**Keywords:** Whiplash injury, Cervical spine pain, Range of movement, Visual Analogue Scale

## Abstract

**Background:**

Whiplash has been suggested to cause chronic symptoms and long term disability. This study was designed to assess long term function after whiplash injury.

**Material & Methods:**

A random sample of patients in the outpatient clinic was interviewed, questionnaire completed and clinical examination performed. Assessment was made of passive cervical range of movement and Visual Analogue Scale pain scores. One hundred and sixty-four patients were divided into four different groups including patients with no whiplash injury but long-standing neck pain (Group A), previous symptomatic whiplash injury and long-standing neck pain (Group B), previous symptomatic whiplash injury and no neck symptoms (Group C), and a control group of patients with no history of whiplash injury or neck symptoms (Group D).

**Results:**

Data was analyzed by performing an Independent samples t-test and ANOVA, with level of significance taken as *p*<0.05. Comparing the four groups using a one-way ANOVA showed a significant difference between the groups (p<0.001). There were significant differences when comparing mean ranges of movement between Group A and Group D, and between Group B and Group D. There was no significant difference between Group C and Group D. similar differences were also seen in the pain scores.

**Conclusion:**

We conclude that osteoarthritis in the cervical spine, and whiplash injury with chronic problems cause a significantly decreased cervical range of movement with a higher pain score. Patients with shorter duration of whiplash symptoms appear to do better in the long-term.

## INTRODUCTION

There are many terms and definitions for whiplash injury including neck or cervical sprain/strain, cervical acceleration-deceleration injury and also Whiplash Associated Disorder (WAD). This injury is best described as an “Injury to one or more elements of the cervical spine that arises from inertial forces being applied to the head in the course of a motor vehicle accident that results in the perception of neck pain” [1]. Accurate epidemiological data is difficult to obtain due to the varying degree of symptoms that passengers involved in rear shunts may suffer. Not all recipients of a whiplash injury may attend the Accident andEmergency department or claim compensation. In the United Kingdom, it was calculated that during 1995 the incidence of whiplash injury was 417 per 100,000 population[2], whereas a study in Canada in 2015 reported a yearly incidence rate was 236 per 100,000 population during the study period [3]. There is evidence from Australia that the incidence of compensable WAD decreased between 2000 and 2009 from 156 to 114 per 100,000 [4].

It is difficult to say whether the incidence of WAD is changing but it nevertheless remains a significant medical, social and financial burden. Richter *et al.* (2000) [5] stated that 10 billion euros had been spent on cervical injuries resulting from rear-end collisions in Europe alone by 2000. Associated costs include physiotherapy, pharmaceuticals, general practitioner, chiropractic, radiology and osteopathy sessions, as well as psychosocial implications and days off work resulting in reduced productivity.

Neck pain is a common symptom throughout life with around 67% of adults complaining of neck pain during their life [6]. Research by Bovim et al showed 34.4% of the population experience neck pain at some time throughout a year, with 13.8% experiencing this pain for over 6 months [7]. Age is also major factor for variability in cervical range of movement (ROM), with a clear decrease in ROM as age increases [8]. Passive ROM has also been shown to be greater than active ROM, therefore any comparisons between two groups would have to include passive measurements [9]. Whiplash has been suggested to cause chronic symptoms and long term disability. This study was designed to assess long term function after whiplash injury.

## METHOD

Following local ethical committee approval, 164 patients waiting in the orthopaedic fracture out-patient clinic waiting area at our institution were interviewed and a questionnaire was completed. The questionnaire asked about previous road traffic accidents resulting in whiplash injury. This was defined for the purpose of this study as an acceleration-deceleration road traffic accident resulting in an acute episode of neck pain. Previous history of cervical spine pathology was also noted and participants that were being seen for any current neck problem including recent fracture and/or dislocation of the cervical spine, spasmodic torticollis and cervical spondylosis were excluded from the study. Patients were asked to mark the question relating to the scoring of neck pain on Visual Analogue Scales (VAS), and the remainder of the questionnaire and examination was completed by the same clinician throughout. Passive cervical ROM was then measured using a goniometer and with the patient seated on the same high backed chair throughout the study. The movements measured were rotation, flexion-extension and lateral flexion ensuring there was no shoulder rotation when measuring rotation and only cervical movement was recorded when measuring flexion-extension and lateral flexion. The totals for the ROM were calculated by adding two results together. These are: left rotation + right rotation = rotation, flexion + extension = flexion-extension, lateral flexion left + lateral flexion right = lateral flexion.

Statistical analysis of the results was carried out by performing an Independent samples t-test and a one-way ANOVA using Statistical Package for Social Sciences (SPSS) 12.0 for Windows.

## RESULTS

In total, 164 patients were interviewed in the study. Within this group the age range was 36-59 years with the average age being 46.8 years. Following completion of the questionnaire, the patients were divided into groups based on their history of road traffic accidents, whiplash injury, cervical osteoarthritis clinically or undiagnosed long-standing neck pain. These consisted of Group A including patients with no previous history of whiplash injury but those that had been diagnosed either with cervical osteoarthritis clinically or undiagnosed long-standing neck pain. For this group, it was assumed that in patients with undiagnosed long-standing neck pain, the symptoms were likely to be due degenerative change consistent with cervical osteoarthritis. Group B included patients with previous symptomatic whiplash injury who had been diagnosed either with cervical osteoarthritis clinically or undiagnosed long-standing neck pain. Group C included patients with previous symptomatic whiplash injury and no history of either cervical osteoarthritis clinically or undiagnosed long-standing neck pain. Group D had never had a whiplash injury. These patients had no history of arthritis in their cervical spine, no history of neck pain and had never had surgery on their cervical spine. This formed the control group.

Group A: Twenty-four patients had either previously diagnosed symptomatic cervical arthritis or undiagnosed long standing neck pain. All of these patients had never suffered a whiplash injury. Mean age was 47.2 years and nine patients in this group were male. All complained of neck pain with an average VAS of 38.9. The results for this group are rotation 134.6º ± 13.9º, flexion-extension 78.3º ± 12.6º, lateral flexion 64.5º ± 14.8º.

Group B: Twenty-eight patients had suffered a symptomatic whiplash injury and had either previously diagnosed symptomatic cervical arthritis or undiagnosed long standing neck pain. Mean age was 46 years and 15 patients were male with an average VAS of 40.7. The results for this group are passive rotation 133.5º ± 14.2º, flexion-extension 82.6º ± 12.3º and lateral flexion 67.2º ± 8.4º. The average time since the accident was 9.5 years with the acute pain after the accident lasting on average 28 weeks. Of this group, 85.7% (n= 24) thought that the whiplash injury had directly affected their neck.

Group C: Twenty-seven patients had suffered a whiplash injury but had no long term cervical discomfort and no history of cervical arthritis. Mean age was 46.3 years and 13 patients were male. The results for this group are passive rotation 152.4º ± 13.1º, flexion-extension 89.6º ± 9.9º and lateral flexion 72.0º ± 9.9º. The average time since the accident was 9.6 years but the acute pain after the accident lasted on average 10.5 weeks, as opposed to 28 weeks for Group B. The number of subjects in this group that thought the whiplash injury had affected their neck was only 18.5% (n=5).

Group D: Of these 85 patients in Group D, 59 patients had never been involved in a road traffic accident and had never had a whiplash injury. These patients had no history of arthritis in their cervical spine or other joints, no history of neck pain and had never had surgery on their cervical spine. This group was labelled Group D1. The mean age of this group was 47.4 years and 26 were male. The results for this group showed rotation of 153.0º ± 10º, flexion extension 91.1º ± 9.2 º and lateral flexion 73.5 º ± 6.9º. Fifteen patients in Group D had been involved in a road traffic accident but did not suffer a whiplash injury and had no previous history of neck surgery or arthritis. This was labelled Group D2. Eleven of these were male with a mean age of 46.4. The results for this group showed rotation 156.9º ± 7.8º, flexion-extension 91.2º ± 8.6º and lateral flexion 73.1º ± 8.5º. Eleven patients in Group D had not suffered a whiplash injury but did have arthritis in joints other than their neck, with no cervical pain or previous cervical surgery. This was labelled Group D3. Four of these patients were male with a mean age of 46.8 years. The results for this group are rotation 156.5º ± 10.6 º flexion-extensions 88.5º 6 ± 6.2º and lateral flexion 74.4º 6 ± 6.9º. As the cervical ROM was similar in Groups D1, D2 and D3 groups and no statistical difference was found on an independent samples t-test, these patients were grouped together to form one larger control group, Group D to allow comparison. This control group consisted of 85 subjects with a mean age of 47.2 years and a range of 36-58 years. The results for this group are rotation 154.1º ± 9.8º, flexion-extension 90.8º ± 8.7º and lateral flexion 73.8 º ± 8.9º.

The mean ROMs for the three movements measured, rotation, flexion-extension and lateral flexion in Groups A, B and C were compared against the control Group D (Table **1**).

The means of the control Group D versus the group with no whiplash injury but with cervical arthritis or long-standing neck pain (Group A) were significant at a *p* level of 0.05 with of rotation *p*<0.0001, flexion-extension *p*<0.0001 and lateral flexion *p*=0.006. The results show that arthritis in the neck decreases the cervical ROM significantly in all movements measured. This was also true when comparing the means of the control Group D against the group with previous symptomatic whiplash injury and cervical arthritis or long-standing neck pain (Group B), where probability values of rotation *p*<0.0001, flexion-extension *p*=0.003 and lateral flexion *p*=0.0001 were found. The results show that symptomatic whiplash injuries with cervical arthritis or long-standing neck pain decreases the cervical ROM significantly in all the directions recorded. However on performing analysis on the means of the control group D against the group with previous symptomatic whiplash injury without cervical arthritis or long-standing neck pain (Group C), the probability values are rotation *p*=0.54, flexion-extension *p*=0.58 and lateral flexion *p*=0.39. These results show that symptomatic whiplash injury with no cervical arthritis or long-standing neck pain does not result in a significant reduction of cervical ROM.

Comparing the four groups using a one-way ANOVA showed a significant difference between the groups (p<0.001). Further post hoc tests show no evidence of a difference between Group A and Group B (p=0.87), nor between groups C & D (p=0.87). The average difference between Group A and Group D is -38.9 (-46.6, -31.1), and between Group B and Group D is -40.7 (-48.05, -33.4) . Similar size differences exist between Group A and Group C -38.9 (-48.3, -29.4) and between Group B and Group C -40.7 (-49.8, -31.6). From these results we can conclude that patients who are asymptomatic have higher ROM levels, regardless of a history of whiplash injury, compared to those who are symptomatic.

The VAS Pain Scores were 0 for Group A and Group B. For Group C and D the values were 38.88 ± 21.53 and 40.71 ± 21.11 respectively. The groups were further analysed by performing an Independent samples t test to compare the VAS Pain Score of the control Group D with patients in Group A (*p*<0.0001), and Group B (*p*<0.0001). There was no statistical difference in the VAS Pain Score between patients in Group D and Group C (*p*=0.76) suggesting that patient who had recovered from their whiplash injury had no residual pain. These results show that the VAS is significantly increased in patients with cervical osteoarthritis or undiagnosed neck pain, whether they have had a previous whiplash injury or not.

## DISCUSSION

This study confirms that cervical movement is indeed reduced in patients with both osteoarthritis and whiplash injuries with prolonged symptoms. However, following a whiplash injury where the acute symptoms settle, cervical movement was found to be similar to the final control group indicating that this group of patients do not suffer long term complications as a result of the whiplash injury. The participants in this study suffered a whiplash injury just over 9.5 years prior to the study and therefore when describing the duration of symptoms the authors accept that this figure may not be completely accurate. However there is an obvious decrease in the duration of symptoms for Group C versus Group B (10.5 weeks *Vs* 28 weeks) and the clear distinction between the two groups is perhaps more important.

There were differences in the range of movement between the currently symptomatic and currently asymptomatic groups regardless of a history of whiplash injury. It would seem unlikely that function is affected by such a relatively small loss in cervical ROM and so cause disability to the patient. Interestingly patients with degenerative cervical neck problems, and patients with a history of whiplash injury and neck symptoms were found to have higher pain scores as compared to patients who have suffered from whiplash injuries with no neck symptoms and control patients.

## CONCLUSION

Degenerative changes in the cervical spine and whiplash injury with chronic problems cause a significantly decreased cervical ROM with a higher pain score. However where whiplash symptoms settle down quickly, patients are not left with any long-term reduction in function or pain. Whiplash injury with residual symptoms does affect the range of movement of the neck in the long term, with statistical significance but this decrease may not be large enough to produce a functional decrease of movement that would cause disability.

## Figures and Tables

**Table 1:** Ranges of movement for the four groups.

Group	N	Mean	Standard deviation	95% Confidence Interval		Minimum	Maximum
				Lower Bound	Upper Bound		
A	24	277.4	32.5	263.7	291.2	215	335
B	28	283.4	24.5	273.8	292.9	218	338
C	27	314.0	28.6	302.7	325.3	251	360
D	85	318.7	22.4	313.9	323.6	245	364
Total	164	305.9	30.7	301.1	310.6	215	364
